# Biomarkers Identify Distinct Biological Signatures of Eccentric Hypertrophy in Elite Athletes: A Sex-Specific Analysis

**DOI:** 10.3390/medicina62071341

**Published:** 2026-07-11

**Authors:** Giuseppe Di Gioia, Armando Ferrera, Pier Giorgio Tiberi, Francesco Raffaele Spera, Viviana Maestrini, Andrea Serdoz, Alessandro Spinelli, Federica Mango, Antonio Pelliccia, Maria Rosaria Squeo

**Affiliations:** 1 Institute of Sports Medicine and Science, National Italian Olympic Committee, Largo Piero Gabrielli, 1, 00197 Rome, Italy; 2 Department of Clinical and Molecular Medicine, Sapienza University of Rome, 00189 Rome, Italy; 3 Department of Clinical, Internal, Anesthesiologic and Cardiovascular Sciences, Sapienza University of Rome, Piazzale Aldo Moro 5, 00185 Rome, Italy

**Keywords:** elite athletes, performance, athlete’s heart, biochemical, gender differences

## Abstract

*Background and Objectives:* Eccentric hypertrophy (EH) represents a common physiological cardiac adaptation in athletes, particularly in endurance disciplines. However, the biological correlates underlying EH and potential sex-specific differences remain poorly understood. This study aimed to investigate clinical, echocardiographic, and biochemical determinants of EH in a large cohort of elite athletes. *Materials and Methods:* We evaluated 2522 elite athletes undergoing pre-participation screening for Olympic competitions. Athletes were classified according to cardiac geometry into EH or normal geometry (NG) based on echocardiographic parameters. Clinical, anthropometric, echocardiographic, and biochemical variables were compared between groups. Univariate and multivariable logistic regression analyses were performed to identify independent predictors of EH. *Results:* EH was present in 601 athletes (23.8%) and was more prevalent in endurance sports (51.1% vs. 14.3%, *p* < 0.0001). Athletes with EH showed greater cardiac chamber dimensions and mass, with preserved systolic and diastolic function. Several biomarkers differed between groups, including higher AST, ALT, CPK, eosinophils, and HDL, and lower creatinine and TSH in EH (all *p* < 0.01). Following multivariable analysis, lower creatinine (OR 0.28, 95% CI 0.14–0.52, *p* < 0.0001), higher AST (OR 1.02, 95% CI 1.02–1.03, *p* < 0.0001), and higher eosinophil count (OR 1.12, 95% CI 1.06–1.20, *p* < 0.0001) independently predicted EH. The model showed moderate discrimination (AUC 0.72). Sex-stratified analyses showed different biomarker associations with EH in men (creatinine and AST) and women (eosinophils and HDL), with similar model performance (AUC 0.71 vs. 0.73). *Conclusions:* EH in elite athletes is associated with distinct biological signatures reflecting multiple physiological pathways. Notably, sex-specific patterns emerge, suggesting different mechanisms underlying cardiac adaptation in male and female athletes.

## 1. Introduction

Regular intensive exercise induces a broad spectrum of cardiovascular adaptations collectively referred to as the “athlete’s heart”. Among these, eccentric hypertrophy (EH) represents one of the most common patterns of physiological cardiac remodeling, particularly in endurance athletes exposed to sustained volume overload. EH is typically characterized by balanced enlargement of left ventricular (LV) cavity dimensions and myocardial mass, while preserving systolic and diastolic function [1,2,3]. Although this phenotype is generally considered a benign adaptation to training, the mechanisms underlying its development remain incompletely understood. A clinically relevant challenge is the differentiation between physiological exercise-induced cardiac remodeling and early or mild forms of pathological dilatation, particularly dilated cardiomyopathy, in highly trained athletes, where phenotypic overlap may occur. Improved characterization of the systemic correlates of physiological adaptation may therefore support more accurate clinical interpretation in borderline cases.

Over the past decades, research in sports cardiology has primarily focused on the structural and functional characterization of cardiac remodeling using electrocardiography and cardiac imaging [4]. In contrast, the biological correlates accompanying physiological remodeling have received considerably less attention [5]. Emerging evidence suggests that exercise-induced cardiac adaptation may reflect a complex systemic response involving vascular regulation, immune modulation, and metabolic efficiency [6,7]. From this perspective, circulating biomarkers may provide indirect information regarding the biological pathways accompanying physiological cardiac remodeling. In particular, markers of muscle metabolism may reflect the systemic consequences of chronic training-induced muscular adaptation, whereas lipid-related, endocrine, and immune biomarkers may capture metabolic efficiency, hormonal regulation, and exercise-induced immunomodulation. Therefore, the assessment of routinely available biochemical parameters may offer additional insight into the systemic mechanisms associated with EH beyond conventional imaging findings.

Previous studies have explored the relationship between selected circulating biomarkers and exercise-induced cardiac adaptation. For example, Di Gioia et al. [8] demonstrated that serum fT3 levels within the euthyroid range were associated with cardiac dimensions and exercise-induced remodeling in a large cohort of elite athletes, suggesting a potential role of thyroid hormones in physiological cardiac adaptation. However, these studies have investigated individual biomarkers or specific biological pathways rather than providing a comprehensive characterization of the biological profile associated with a distinct remodeling phenotype such as EH.

In addition, growing evidence suggests that sex substantially influences cardiovascular adaptation to exercise [9]. Male and female athletes differ in cardiac morphology, hormonal profile, substrate utilization, autonomic regulation, and immune responses [10]. Nevertheless, most studies investigating athlete’s heart have predominantly included male populations, and potential sex-specific biological determinants of physiological remodeling remain poorly understood.

Thus, the aim of the present study was to investigate the clinical, echocardiographic, and biochemical correlates of EH in a large cohort of elite athletes. We hypothesized that: (1) EH in elite athletes would be associated with a distinct biological profile beyond conventional echocardiographic characteristics and (2) sex-specific differences would exist in the biological correlates of physiological cardiac remodeling.

## 2. Materials and Methods

The Institute of Sport Medicine and Science in Rome is a specialized institution overseen by the Italian National Olympic Committee whose primary responsibility is to conduct medical evaluations of athletes selected to participate in international events. The research methodology employed in this investigation underwent scrutiny and approval by the Ethical Committee of USL Rome 1—Sapienza University of Rome (date of approval 25 September 2024; IRB number 0851/2024).

**Figure 1:**
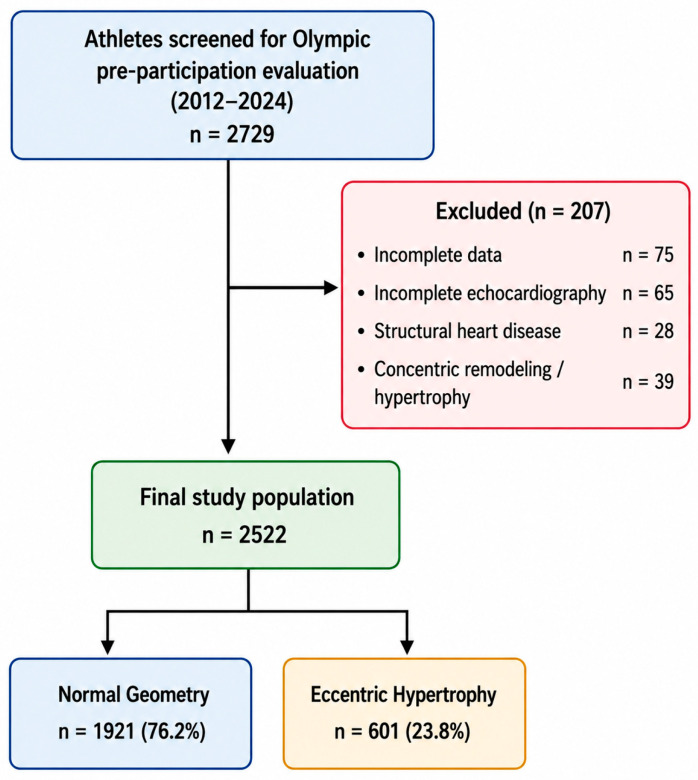
Flow diagram illustrating the selection of the study population. Between 2012 and 2024, 2729 elite athletes underwent Olympic pre-participation cardiovascular evaluation at the Institute of Sports Medicine and Science. A total of 207 athletes were excluded because of incomplete clinical or laboratory data (n = 75), incomplete echocardiographic assessment (n = 65), structural heart disease (n = 28), or concentric left ventricular remodeling/hypertrophy (n = 39). The final study cohort consisted of 2522 elite athletes, including 1921 (76.2%) with normal left ventricular geometry and 601 (23.8%) with eccentric hypertrophy.

We enrolled 2522 elite athletes participating in the Italian National Olympic Program who underwent comprehensive pre-participation cardiovascular evaluation at the Institute of Sport Medicine and Science prior to major international competitions and successive Summer Olympic cycles (London 2012, Rio 2016, Tokyo 2020 and Paris 2024). Athletes were referred by their respective National Sport Federations as part of the Italian Olympic selection pathway. Athletes with incomplete clinical, biochemical or echocardiographic data, known cardiovascular disease, structural cardiac abnormalities, concentric remodeling, or concentric hypertrophy were excluded from the final analysis (Figure 1).

Athletes participated in 42 disciplines, divided into the skill, power, mixed and endurance categories according to European classification. For disciplines not specifically included in the ESC classification, categorization was performed according to the recommendations of the Italian COCIS (Cardiological Guidelines for Competitive Sports Eligibility) [11]. The athletes represented a wide range of sporting disciplines. Skill-based sports included archery, skeet and target shooting, golf, park and street skateboarding, equestrian events, table tennis, and sailing. Power disciplines comprised weightlifting, diving, artistic swimming, taekwondo, athletics (events under 800 m), boxing, artistic gymnastics, judo, Greco-Roman wrestling, climbing, surfing, swimming (events under 400 m), kickboxing, and karate, as well as BMX and mountain biking. Mixed included volleyball, beach volleyball, basketball, rugby, water polo, fencing, tennis, dancing, rhythmic gymnastics, and badminton, and, finally, canoeing, rowing, marathon-swimming, pentathlon, marathon running, cycling and triathlons were classified as endurance sports.

Athletes underwent a comprehensive, multidisciplinary pre-participation screening (PPS), unchanged throughout the study period (2012–2024), which included a complete physical examination, a full panel of blood tests, resting electrocardiography (ECG) and transthoracic echocardiography (TTE). Blood test analysis was performed in all athletes within the same laboratory, at the same morning hours (between 7.30 and 9 am), after an overnight fast and at least 24 h after the last training session, with an aseptic technique from a vein in the antecubital fossa. The following biochemical parameters were assessed: full blood count, ferritin, transferrin, iron, potassium, calcium, magnesium, urate, creatinine, aspartate amino-transaminase (AST), alanine aminotransferase (ALT), total cholesterol (TC), low-density lipoprotein (LDL), high-density lipoprotein (HDL), triglycerides (TG), glucose, C-reactive protein (CRP), thyroid-stimulating hormone (TSH), ft3, ft4, vitamin D, total (TB) and direct bilirubin (DB).

The echocardiographic evaluation was conducted on participants while they were at rest, positioned in the left lateral decubitus position. Ultrasound data acquisition was performed utilizing a GE Vivid E9 ultrasound system equipped with a 4Vc phased array probe (GE Healthcare Vingmed Ultrasound AS, Horten, Norway). All echocardiographic examinations were performed at the Institute of Sport Medicine and Science using the same echocardiographic platform (GE Vivid E9, GE Healthcare, Horten, Norway) and a standardized acquisition protocol throughout the study period.

A comprehensive 2D echocardiographic study was carried out, in which cardiac images were captured in various cross-sectional planes employing established transducer positions. Conventional echocardiography was conducted according to American Society of Echocardiography (ASE) guidelines and the European Association of Cardiovascular Imaging [12,13]. LV wall thickness was measured in the parasternal long-axis view. Left ventricular ejection fraction (LVEF) and left atrial volume index (LAVi) were determined using a modified Simpson’s method. The peak early diastolic velocity of LV inflow (E velocity), the late atrial diastolic velocity of LV inflow (A velocity), and the peak early diastolic velocity at the septal corner of the mitral annulus (e′) were recorded from the apical four-chamber view [12,13]. Relative wall thickness (RWT) and LV mass (LVM) were therefore calculated in order to classify LV geometry in normal geometry (NG), defined as LVM ≤ 115 g/m^2^ in males or ≤ 95 g/m^2^ in females, and RWT ≤ 0.42 and EH as LVM > 115 g/m^2^ in males or > 95 g/m^2^ in females. LVM was calculated using the Devereux-modified ASE formula [LVM = 0.8 × {1.04 × [(IVSd + LVIDd + PWTd)^3^ − (LVIDd)^3^]} + 0.6 g], according to the recommendations of the American Society of Echocardiography and the European Association of Cardiovascular Imaging. LVM was indexed to body surface area (LVM/BSA) [14].

Inter- and intra-observer reproducibility of echocardiographic measurements has been previously established in our laboratory using the same standardized imaging protocol, echocardiographic equipment, and experienced core team of sports cardiologists, as reported in previous methodological publications [15,16,17].

Athletes with concentric hypertrophy and concentric remodeling were excluded from the study.

## 3. Statistical Analysis

Before statistical analysis, data completeness was assessed for all study variables. Athletes with missing clinical, biochemical, or echocardiographic data were excluded from the analyses (complete-case analysis); therefore, no imputation of missing values was performed. Continuous variables are expressed as mean ± standard deviation (SD), while categorical variables are reported as absolute numbers and percentages. The distribution of continuous variables was assessed for normality using the Shapiro–Wilk test and visual inspection of histograms. Given the large sample size, parametric tests were applied. Comparisons between athletes with EH and NG were performed using the independent samples Student’s *t*-test for continuous variables and the χ^2^ test for categorical variables. Univariate logistic regression analysis was conducted to evaluate the association between each biomarker and the presence of EH. Results are reported as odds ratios (ORs) with corresponding 95% confidence intervals (CIs). The explanatory power of each variable was assessed using the Nagelkerke R^2^. The multivariable model was constructed using a predefined biological framework. Candidate variables were selected based on biological plausibility, evidence from the previous literature, and their association with EH at univariate analysis. Variables representing overlapping physiological domains were evaluated for multicollinearity, and only the most informative marker was retained to preserve model parsimony and biological interpretability. Multicollinearity among variables was assessed using the variance inflation factor (VIF), with a threshold of >5 indicating significant collinearity. Among markers of muscle metabolism, AST, ALT, and CPK showed evidence of multicollinearity; therefore, ALT and CPK were excluded, while AST was retained based on its stronger association with EH and lower variability. All remaining variables demonstrated acceptable collinearity (VIF < 2.0). To preserve model parsimony and avoid overfitting, variables with weaker associations at univariate analysis were not included in the final model. The final multivariable logistic regression model included age, smoking status, creatinine, AST, eosinophil count, HDL cholesterol, and TSH. Separate sex-stratified multivariable analyses were performed to explore potential differences in determinants of EH between male and female athletes. Model discrimination was evaluated using the area under the receiver operating characteristic curve (AUC). Sensitivity and specificity were determined at the optimal Youden index cut-off. Internal validation was performed using a 10-fold cross-validation procedure to assess model stability.

Sex-stratified analyses were performed using the same covariates retained in the overall multivariable model to facilitate direct comparison of biomarker associations between men and women. These analyses were intended to explore potential sex-related differences in the strength of associations rather than to derive independent sex-specific prediction models. Formal effect modification by sex was subsequently assessed by including sex × biomarker interaction terms in the multivariable logistic regression models. The statistical significance of each interaction term was used to determine whether the association between each biomarker and EH differed between men and women.

Statistical significance was defined as a two-sided *p*-value < 0.05. Statistical analysis was performed with STATA Statistics software for Windows (SE, version 17).

## 4. Results

A total of 2522 competitive athletes were included in the study. The mean age of the overall population was 25.9 ± 5.1 years. The study population comprised 1381 men (54.8%) and 1141 women (45.2%). Overall, 104 athletes (4.1%) were Afro-Caribbean. Athletes were distributed across skill (n = 630), power (n = 640), mixed (n = 670), and endurance (n = 582) disciplines. Among them, 601 (23.8%) presented EH, while 1921 (76.2%) showed NG. A comparison of general, clinical and anthropometric characteristics between athletes with EH and NG is listed in Table 1. Athletes with EH were slightly older than those with NG (26.5 ± 4.7 vs. 25.5 ± 5.3 years, *p* < 0.0001). The distribution of sport disciplines differed significantly between groups (all *p* < 0.0001), with EH being markedly more prevalent among endurance athletes (51.1% vs. 14.3%), while skill, power, and mixed disciplines were more frequently represented in athletes with NG. No significant differences were observed between groups in terms of sex distribution (47.3% vs. 44.6% female, *p* = 0.238) or ethnicity, including Afro-Caribbean athletes (3.8% vs. 4.2%, *p* = 0.763). The distribution of sport categories according to sex within the EH and NG groups is reported in Supplementary Table S1. No significant differences in sport category distribution were observed between male and female athletes in either the EH group (*p* = 0.143) or the NG group (*p* = 0.416). Anthropometric parameters, including height, body weight, and BMI, were comparable between groups. Similarly, systolic and diastolic blood pressure values did not differ significantly between EH and NG athletes. The prevalence of a family history of CAD was similar between groups (*p* = 0.905). In contrast, smoking was significantly less frequent among athletes with EH compared to those with NG (4.0% vs. 9.6%, *p* < 0.0001).

Echocardiographic characteristics of athletes with EH and NG are reported in Table 2. Athletes with EH showed significantly larger left ventricular dimensions compared to those with NG, including higher indexed end-diastolic and end-systolic diameters and volumes (all *p* < 0.0001). LV wall thicknesses were also significantly increased in the EH group (both *p* < 0.0001). Despite these structural differences, LVEF was preserved and similar between groups (*p* = 0.616). Atrial dimensions were significantly larger in athletes with EH, with higher LA and LAVI (both *p* < 0.0001). RV dimensions were also significantly greater in the EH group, including indexed RV outflow tract diameter and RVEDD, as well as RV areas (all *p* < 0.0001). Right ventricular systolic function, assessed by FAC, was comparable between groups (*p* = 0.933), while TAPSE was slightly higher in EH athletes (*p* < 0.0001). Diastolic function analysis revealed no differences in early diastolic filling (E wave), but a significantly lower A wave velocity in EH athletes, resulting in a higher E/A ratio (both *p* < 0.0001). Tissue Doppler parameters (E′, E/E′, and S′) were similar between groups (all *p* > 0.05).

Significant differences in several biochemical parameters were observed (Table 3). Athletes with EH exhibited higher levels of biomarkers related to muscle metabolism, including AST, ALT, and CPK (all *p* < 0.0001). In addition, eosinophil count was significantly increased in the EH group compared to the NG athletes (*p* < 0.0001). Markers of metabolic profile also differed between groups. HDL cholesterol levels were higher in athletes with EH (*p* < 0.0001), while triglyceride levels were slightly lower (*p* = 0.009). Creatinine levels were significantly lower in the EH group (*p* < 0.0001). Endocrine parameters showed modest differences, with lower TSH levels in EH athletes (*p* = 0.002) and higher vitamin D levels (*p* = 0.008). A small but significant increase in mean corpuscular volume (MCV) was also observed (*p* = 0.001). No significant differences were observed for the remaining hematological and biochemical parameters, including: RBC (*p* = 0.072), Hb (*p* = 0.063), HCT (*p* = 0.029), MCH (*p* = 0.214), MCHC (*p* = 0.331), RDW-SD (*p* = 0.412), RDW-CV (*p* = 0.287), platelets (*p* = 0.198), WBC (*p* = 0.155), neutrophils (*p* = 0.042), lymphocytes (*p* = 0.118), monocytes (*p* = 0.065), basophils (*p* = 0.221), reticulocytes (*p* = 0.309), total proteins (*p* = 0.274), glucose (*p* = 0.191), urea (*p* = 0.133), iron (*p* = 0.147), TC (*p* = 0.023), LDL (*p* = 0.084), bilirubin (*p* = 0.265), GGT (*p* = 0.173), sodium (*p* = 0.201), potassium (*p* = 0.156), calcium (*p* = 0.082), magnesium (*p* = 0.119), transferrin (*p* = 0.046), CRP (*p* = 0.188), coagulation parameters (*p* > 0.10), ferritin (*p* = 0.097), FT3 (*p* = 0.144), FT4 (*p* = 0.167) and cortisol (*p* = 0.132).

In the univariate logistic regression analysis, all selected biomarkers were significantly associated with EH (Table 4). Creatinine showed the strongest association, with lower values being markedly associated with a higher likelihood of EH (OR 0.30, 95% CI 0.24–0.38, *p* < 0.0001). Among markers of muscle metabolism, AST, ALT and CPK were positively associated with EH. Eosinophil count was also significantly associated with EH (OR 1.15, 95% CI 1.10–1.21, *p* < 0.0001). In addition, HDL cholesterol levels showed a positive association, while TG was inversely associated with EH. Among endocrine and hematological variables, TSH was inversely associated with EH, whereas MCV and vitamin D levels were positively associated.

**Figure 2:**
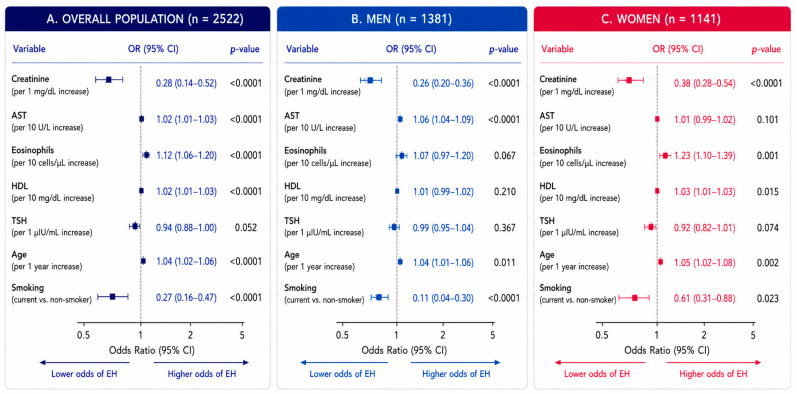
Forest plots of multivariable logistic regression analyses for eccentric hypertrophy. Forest plots showing the odds ratios (ORs) and 95% confidence intervals (CIs) for the independent associations between selected biochemical biomarkers and eccentric hypertrophy (EH) in the overall study population (**A**), male athletes (**B**), and female athletes (**C**). The overall model was adjusted for age, smoking status, creatinine, AST, eosinophil count, HDL cholesterol, and TSH. Sex-stratified models were adjusted for the same covariates. Abbreviations: AST, aspartate aminotransferase; CI, confidence interval; EH, eccentric hypertrophy; HDL, high-density lipoprotein cholesterol; OR, odds ratio; TSH, thyroid-stimulating hormone.

In order to create a multivariable model, collinearity among candidate variables was assessed using VIF. Markers of muscle metabolism, including AST, ALT, and CPK, showed evidence of multicollinearity, with VIF values of approximately 2.8 for AST, 5.6 for ALT, and 6.9 for CPK. Based on these findings and considering their shared physiological domain, ALT and CPK were excluded from the multivariable model, while AST was retained due to its stronger association with EH and lower variability. All remaining variables showed acceptable levels of collinearity, with VIF values below 2.0 (creatinine: 1.2; eosinophils: 1.1; HDL: 1.5; triglycerides: 1.8; TSH: 1.3; vitamin D: 1.2; and MCV: 1.4). Given their weaker associations at univariate analysis and to preserve model parsimony, TG, MCV, and vitamin D were not included in the final multivariable model. Following multivariable logistic regression analysis (Table 5, Figure 2), creatinine, AST, and eosinophil count emerged as independent predictors of EH. Lower creatinine levels (OR 0.28, 95% CI 0.14–0.52, *p* < 0.0001), higher AST (OR 1.02, 95% CI 1.02–1.03, *p* < 0.0001), and higher eosinophils (OR 1.12, 95% CI 1.06–1.20, *p* < 0.0001) were significantly associated with EH. Also, HDL showed a positive association (OR 1.02, 95% CI 1.01–1.03, *p* < 0.0001), while TSH did not retain statistical significance in the final model. After additional adjustment for age and smoking status, the overall pattern of independent biomarker associations remained substantially unchanged, confirming the robustness of the multivariable model. The final model demonstrated moderate discriminative ability, with an area under the curve (AUC) of 0.72. The moderate discriminative performance of the model suggests that EH is not driven by a single biological pathway, but rather may reflect a complex, multifactorial adaptation. At the optimal Youden cut-off, the model achieved a sensitivity of 68.3% and a specificity of 64.7%. Internal validation using 10-fold cross-validation yielded a comparable AUC (0.69), supporting the stability and robustness of the model.

As a sensitivity analysis, the multivariable logistic regression model was additionally adjusted for sport discipline (endurance vs. non-endurance). As expected, participation in endurance sports was strongly associated with the presence of EH (OR 7.74, 95% CI 6.10–9.82; *p* < 0.0001). Importantly, the independent associations between EH and lower creatinine (OR 0.38, 95% CI 0.19–0.74; *p* = 0.005), higher AST (OR 1.01, 95% CI 1.002–1.020; *p* = 0.021), higher eosinophil count (OR 1.09, 95% CI 1.04–1.14; *p* < 0.001), higher HDL cholesterol (OR 1.01, 95% CI 1.003–1.017; *p* = 0.005), and lower TSH (OR 0.86, 95% CI 0.77–0.95; *p* = 0.005) remained statistically significant after adjustment for sport discipline.

When stratified by sex, distinct patterns emerged. In male athletes, creatinine and AST were the only independent predictors of EH.

Lower creatinine levels were strongly associated with EH (OR 0.26, 95% CI 0.20–0.36, *p* < 0.0001), as well as higher AST values (OR 1.06, 95% CI 1.024–1.09, *p* < 0.0001), suggesting that markers related to skeletal muscle metabolism showed a stronger independent association with EH in male athletes. Other variables, including eosinophils (OR 1.07, 95% CI 0.97–1.20, *p* = 0.067), HDL (OR 1.01, 95% CI 0.99–1.02, *p* = 0.210), and TSH (OR 0.99, 95% CI 0.95–1.04, *p* = 0.367), were not independently associated with EH in males. In female athletes, eosinophil count and HDL levels were independently associated with EH. Higher eosinophil values (OR 1.23, 95% CI 1.10–1.39, *p* = 0.001) and higher HDL levels (OR 1.03, 95% CI 1.01–1.03, *p* = 0.015) were associated with EH, together with lower creatinine levels (OR 0.38, 95% CI 0.28–0.54, *p* < 0.0001). AST did not retain statistical significance in females (OR 1.01, 95% CI 0.99–1.02, *p* = 0.101), nor did TSH (OR 0.92, 95% CI 0.82–1.01, *p* = 0.074). Model performance was comparable between sexes, with AUC values of 0.71 in males and 0.73 in females.

Formal interaction testing identified significant sex interactions for AST (*p* < 0.001), eosinophils (*p* = 0.042), HDL cholesterol (*p* = 0.037), and TSH (*p* = 0.014), whereas no significant interaction was observed for creatinine (*p* = 0.643) (Supplementary Table S2).

## 5. Discussion

In this large cohort of elite athletes, EH was strongly associated with endurance disciplines, in keeping with the established hemodynamic model of athlete’s heart, whereby sustained exposure to high-volume dynamic exercise leads to chronic increases in preload, venous return, and cardiac output, resulting in balanced enlargement of cardiac chambers with preserved systolic function [1,4].

However, EH should not be interpreted merely as a marker of endurance training, but rather as a phenotypic pattern of physiological cardiac adaptation to chronic athletic conditioning. Although more prevalent in endurance disciplines, EH was also observed in athletes participating in skill-, power-, and mixed-type sports. This likely reflects the evolution of contemporary elite training programs, in which substantial volumes of high-intensity aerobic conditioning are incorporated even in disciplines traditionally considered predominantly technical or strength-based. Consequently, the biological profile identified in the present study should be interpreted as reflecting the systemic adaptations accompanying this phenotypic pattern of cardiac remodeling rather than the exclusive effects of endurance exercise.

Beyond this classical structural framework, multivariable analysis identified a distinct and independent biological signature of EH, characterized by lower creatinine levels together with higher AST values and increased eosinophil counts, suggesting that physiological cardiac remodeling is accompanied by coordinated systemic adaptations involving plasma volume regulation, skeletal muscle metabolism, and immune activity.

Among these variables, AST was independently associated with EH. In athletes, mild elevations of AST are commonly interpreted as the consequence of increased skeletal muscle turnover and exercise-induced muscular remodeling rather than hepatocellular injury [18]. Repetitive training exposure, particularly in endurance disciplines, induces continuous cycles of muscle microinjury and repair associated with increased enzymatic release. Therefore, the association between AST and EH likely reflects the parallel adaptation of skeletal muscle and cardiovascular systems to chronic exercise stimuli [19]. Notably, this association remained significant only in male athletes, suggesting that muscle-related metabolic adaptation may represent a predominant component of physiological remodeling in men. An alternative explanation should also be considered. Although blood samples were collected according to a standardized protocol requiring at least 24 h without training, AST concentrations may still have been influenced by incomplete recovery from recent exercise, particularly in endurance athletes, in whom muscle enzyme elevations can persist beyond 24 h. Therefore, part of the observed association may reflect chronic training exposure and recovery status in addition to physiological cardiac remodeling. This possibility should be taken into account when interpreting the present findings.

The inverse association between creatinine levels and EH may initially appear counterintuitive, given the established relationship between creatinine production and skeletal muscle mass. One possible explanation is that endurance-trained athletes frequently exhibit plasma volume expansion, enhanced circulatory efficiency [20,21], and relative hemodilution, which may contribute to lower circulating creatinine concentrations despite preserved or increased lean body mass. In this context, lower creatinine levels may be consistent with an indirect marker of the hemodynamic and volume-related adaptations accompanying endurance training rather than a simple surrogate of muscle quantity. The consistency of this association across both sexes is consistent with the hypothesis that plasma volume adaptation represents a central component of physiological EH. However, because plasma volume was not directly measured, this interpretation remains speculative. Alternative explanations, including differences in hydration status, nutritional habits, sport-specific body composition, and skeletal muscle metabolism, cannot be excluded. Therefore, lower creatinine should be interpreted as a biological correlate of the EH phenotype rather than a direct marker of plasma volume expansion.

Another important finding was the independent association between eosinophil count and EH. Although eosinophils are traditionally linked to allergic and inflammatory responses, emerging evidence suggests that they may also participate in tissue repair, angiogenesis, extracellular matrix regulation, and modulation of local immune homeostasis [22,23]. Exercise training itself is increasingly recognized as a potent immunomodulatory stimulus capable of inducing complex adaptive changes in both innate and adaptive immunity [24]. While these observations provide a biologically plausible framework for interpreting our findings, the present study did not directly assess these pathways. Therefore, the observed association should be interpreted as identifying eosinophils as a biological correlate of the EH phenotype rather than demonstrating a specific mechanistic role in exercise-induced cardiac remodeling. The modest increase in eosinophil count observed in athletes with EH, while remaining within the normal physiological range, may reflect a subtle systemic biological correlate of physiological cardiac remodeling rather than an activated inflammatory or immune response.

One of the most novel aspects of the present study is the identification of sex-specific biological signatures associated with EH. In male athletes, EH was independently associated only with lower creatinine and higher AST values, whereas eosinophils and HDL did not retain statistical significance. This pattern suggests that physiological remodeling in men may be more strongly linked to musculoskeletal and hemodynamic adaptation. Such an interpretation is biologically plausible considering the greater absolute skeletal muscle mass, higher exercise-related metabolic load, and different hormonal milieu typically observed in male athletes.

Conversely, in female athletes, eosinophil count and HDL levels emerged as independent correlates of EH, whereas AST lost statistical significance. Although HDL cholesterol remained independently associated with EH after multivariable adjustment, the relatively small effect size suggests that this finding contributes to the overall biological characterization of physiological eccentric remodeling rather than representing a clinically relevant discriminator at the individual level. These findings suggest that physiological remodeling in women may be relatively less dependent on muscle turnover and more closely associated with metabolic and immune-regulatory pathways. This interpretation is consistent with known sex-related differences in substrate utilization and cardiovascular physiology. Female athletes rely proportionally more on lipid metabolism during prolonged exercise, exhibit higher HDL concentrations, and demonstrate distinct immune and inflammatory responses compared with men [25,26]. In addition, estrogen-related signaling influences endothelial function, lipid handling, mitochondrial metabolism, and immune regulation, potentially contributing to different patterns of exercise adaptation [27]. The observed sex-specific associations in the present study support the concept that athlete’s heart should not be interpreted through a uniform biological model across sexes.

Although the absolute differences observed for biomarkers such as HDL cholesterol, triglycerides, and vitamin D were modest, they may reflect subtle systemic adaptations accompanying physiological cardiac remodeling in elite athletes. Therefore, their significance should be interpreted primarily within the biological context of the study rather than as isolated clinical markers.

In contrast, women exhibit a greater reliance on lipid oxidation during prolonged exercise, enhanced mitochondrial efficiency, and a more favorable lipoprotein profile, characterized by higher HDL concentrations. Estrogen has been shown to modulate lipid metabolism, endothelial function, nitric oxide bioavailability, and vascular compliance, factors that may influence both systemic adaptation to exercise and cardiac remodeling. Furthermore, accumulating evidence suggests that females display distinct immune responses to chronic exercise, with differences in cytokine production, leukocyte trafficking, and tissue repair mechanisms. The independent association between eosinophil count and EH observed in women may therefore reflect a greater contribution of immune-regulatory pathways to physiological cardiac adaptation.

Taken together, these observations support the hypothesis that EH may represent the final common phenotype of multiple adaptive mechanisms, whose relative contribution may differ between male and female athletes.

The moderate discriminative performance of the multivariable model (AUC ≈ 0.72) should not be interpreted as a limitation of the biological relevance of the findings. Rather, it reflects the continuous and multidimensional nature of physiological cardiac remodeling, which does not conform to a binary phenotype but rather may represent a graded adaptive spectrum to chronic exercise exposure. In this context, circulating biomarkers provide a robust and biologically coherent signal of dominant adaptive axes rather than a complete reconstruction of all contributing determinants.

Accordingly, the observed AUC should be interpreted as indicating that a relatively small set of circulating biomarkers captures a meaningful component of the biological phenotype underlying physiological EH, while acknowledging that residual variability reflects inter-individual differences in training load, genetic background, and peripheral physiological responses that are not fully captured by circulating measures alone.

From a clinical perspective, the identification of biological signatures associated with EH may contribute to a more integrated understanding of athlete’s heart. Current evaluation of physiological remodeling relies predominantly on imaging findings, while circulating biomarkers are generally interpreted only in the context of pathology or overtraining. Our findings suggest that selected biomarkers may also reflect adaptive physiological pathways associated with exercise-induced remodeling.

Rather than providing a diagnostic tool, the present findings contribute to the biological characterization of physiological EH in elite athletes. Whether this biomarker profile has incremental clinical value for differentiating physiological remodeling from pathological conditions remains to be established in prospective externally validated studies.

## 6. Limitations

Several limitations should be acknowledged. First, the cross-sectional design precludes any causal inference between biomarkers and EH. The observed associations may reflect consequences rather than determinants of cardiac remodeling. Second, training load, duration of athletic career, and cardiorespiratory fitness (e.g., VO_2_ max) were not available. These variables are well-recognized determinants of physiological cardiac remodeling. However, the present study was conducted exclusively in elite Olympic athletes, representing a highly selected and relatively homogeneous population that had undergone many years of high-level athletic training before Olympic selection. Nevertheless, the absence of individual training metrics precludes assessment of their independent contribution to the observed biological profile and should therefore be considered when interpreting our findings. Third, although blood samples were collected under standardized conditions, biomarker levels may still be influenced by acute or recent training, hydration status, and inter-individual variability. In particular, the interpretation of creatinine levels is limited by the lack of direct assessment of plasma volume or renal function. Furthermore, nutritional habits, body composition, and other sport-specific physiological characteristics were not systematically collected and may also have influenced circulating creatinine concentrations. Fourth, AST is not a tissue-specific marker, and its elevation cannot be exclusively attributed to skeletal muscle metabolism. Similarly, eosinophil count represents a non-specific immune marker, and its mechanistic role in cardiac remodeling remains speculative. Fifth, the study did not include advanced imaging parameters, such as myocardial strain or tissue characterization, which could provide further insight into the functional correlates of EH. Furthermore, information regarding dietary habits, nutritional intake, hydration status, and the use of supplements was not systematically available. These factors may influence several biochemical parameters, including vitamin D, lipid profile, iron metabolism markers, and muscle-related enzymes, and therefore represent potential unmeasured confounders. Future prospective studies incorporating detailed nutritional assessments may help further clarify the biological determinants of exercise-induced cardiac remodeling.

As a result, although multiple circulating biomarkers were evaluated, no formal correction for multiple comparisons was applied because the study was hypothesis-driven and the primary conclusions were based on multivariable analyses rather than individual univariate associations. Nevertheless, the possibility of type I error cannot be completely excluded, and the identified biomarker associations should be confirmed in independent cohorts.

Finally, the cohort consisted exclusively of elite athletes, limiting the generalizability of the findings to recreational or non-athletic populations.

## 7. Conclusions

This study supports the hypothesis that EH in elite athletes is associated with a distinct biological profile extending beyond conventional cardiac structural adaptation. Lower creatinine levels together with higher AST and eosinophil counts emerged as independent correlates of EH, suggesting that EH is associated with biological signatures involving hemodynamic, metabolic, muscular, and immune-regulatory pathways in physiological cardiac remodeling. Furthermore, our findings support the hypothesis of sex-specific mechanisms underlying athlete’s heart. While EH in male athletes was predominantly associated with markers of muscle-related adaptation, female athletes exhibited a stronger association with immune and metabolic biomarkers, particularly eosinophils and HDL cholesterol.

These results provide novel evidence that physiological cardiac remodeling represents part of a broader systemic adaptation to long-term exercise training and that the biological pathways associated with EH differ between sexes. Such findings contribute to a more individualized and biologically integrated interpretation of the athlete’s heart.

## Figures and Tables

**Table 1:** General, clinical and anthropometric characteristics of athletes with EH vs. NG.

Variable	EH (n = 601)	NG (n = 1921)	*p*-Value
Age (years)	26.5 ± 4.7	25.5 ± 5.3	<0.0001
Skill sport	18 (3.0%)	612 (31.9%)	<0.0001
Power sport	124 (20.6%)	516 (26.9%)	<0.0001
Mixed sport	152 (25.3%)	518 (27%)	0.008
Endurance sport	307 (51.1%)	275 (14.3%)	<0.0001
Female, n (%)	284 (47.3%)	857 (44.6%)	0.238
Afro-Caribbean, n (%)	23 (3.8%)	81 (4.2%)	0.763
Height (cm)	177.0 ± 10.6	177.0 ± 11.1	0.954
Weight (kg)	73.0 ± 15.2	73.6 ± 14.8	0.353
BMI (kg/m^2^)	23.0 ± 3.0	23.3 ± 3.1	0.090
Systolic BP (mmHg)	112.3 ± 11.6	112.3 ± 11.1	0.935
Diastolic BP (mmHg)	69.3 ± 8.2	69.6 ± 19.0	0.692
Family history of CAD, n (%)	147 (24.6%)	479 (25.0%)	0.905
Smoking	24 (4.0%)	184 (9.6%)	<0.0001

Abbreviations: BMI, body mass index; BP, blood pressure; CAD, coronary artery disease; EH, eccentric hypertrophy; NG, normal geometry.

**Table 2:** Echocardiographic characteristics (EH vs. NG).

Variable	EH (n = 601)	NG (n = 1921)	*p*-Value
LVEDD/BSA (mm)	30.0 ± 3.8	27.5 ± 2.3	<0.0001
LVESD/BSA (mm)	18.4 ± 2.8	17.0 ± 2.0	<0.0001
LVEDV/BSA (mL)	80.3 ± 18.3	66.4 ± 14.1	<0.0001
LVESV/BSA (mL)	29.4 ± 8.5	24.1 ± 6.5	<0.0001
IVS (mm)	10.3 ± 1.0	9.0 ± 1.0	<0.0001
PWT (mm)	10.0 ± 1.0	8.6 ± 1.1	<0.0001
EF (%)	63.6 ± 5.3	63.5 ± 5.4	0.616
RWT	0.40 ± 0.06	0.37 ± 0.07	<0.0001
LVM/BSA (g/m^2^)	119.1 ± 16.7	86.0 ± 14.3	<0.0001
LA (mm)	38.2 ± 4.4	34.7 ± 4.3	<0.0001
LAVI (ml/m^2^)	25.9 ± 7.5	20.2 ± 6.1	<0.0001
RA (mm)	26.0 ± 8.8	21.1 ± 9.7	<0.0001
RVOT LAX/BSA (mm)	17.1 ± 2.8	15.4 ± 2.3	<0.0001
RVEDD/BSA (mm)	21.6 ± 2.5	19.8 ± 2.5	<0.0001
RVEDA (mm^2^)	23.9 ± 5.5	21.4 ± 5.0	<0.0001
RVESA (mm^2^)	12.7 ± 3.2	11.4 ± 3.0	<0.0001
FAC (%)	46.6 ± 7.9	46.5 ± 7.0	0.933
TAPSE (mm)	27.3 ± 4.2	26.0 ± 4.2	<0.0001
E wave (cm/sec)	82.9 ± 14.9	82.9 ± 16.6	0.945
A wave (cm/sec)	59.5 ± 24.8	69.3 ± 26.1	<0.0001
E/A ratio	1.6 ± 0.6	1.4 ± 0.7	<0.0001
E′ (m/sec)	12.1 ± 2.1	12.3 ± 2.5	0.058
E/E′	7.0 ± 1.5	6.9 ± 1.6	0.166
S′ (m/sec)	8.2 ± 1.7	8.3 ± 2.0	0.339

Abbreviations: A, late diastolic transmitral flow velocity; E, early diastolic transmitral flow velocity; E′, early diastolic mitral annular velocity; E/E′, ratio of early transmitral flow velocity to early diastolic mitral annular velocity; EF, ejection fraction; FAC, fractional area change; IVS, interventricular septum; LA, left atrium; LAVI, left atrial volume index; LVEDD/BSA, left ventricular end-diastolic diameter indexed to body surface area; LVEDV/BSA, left ventricular end-diastolic volume indexed to body surface area; LVESD/BSA, left ventricular end-systolic diameter indexed to body surface area; LVESV/BSA, left ventricular end-systolic volume indexed to body surface area; LVM/BSA, left ventricular mass indexed to body surface area; NG, normal geometry; PWT, posterior wall thickness; RA, right atrium; RWT, relative wall thickness; RVEDA, right ventricular end-diastolic area; RVEDD/BSA, right ventricular end-diastolic diameter indexed to body surface area; RVESA, right ventricular end-systolic area; RVOT LAX/BSA, right ventricular outflow tract diameter in long-axis view indexed to body surface area; S′, systolic mitral annular velocity; TAPSE, tricuspid annular plane systolic excursion.

**Table 3:** Significant differences in biomarkers (EH vs. NG).

Variable	EH (n = 601)	NG (n = 1921)	*p*-Value
Eosinophils, 10^3^/uL	2.0 ± 1.3	1.7 ± 1.2	<0.0001
Creatinine, mg/dL	0.93 ± 0.17	0.99 ± 0.18	<0.0001
AST, U/L	28.4 ± 11.9	24.4 ± 10.2	<0.0001
ALT, U/L	23.2 ± 11.3	20.6 ± 9.8	<0.0001
CPK, U/L	310.1 ± 214.5	275.9 ± 198.3	<0.0001
HDL, mg/dL	68.7 ± 17.2	64.4 ± 16.8	<0.0001
TSH, µIU/mL	2.0 ± 1.2	2.3 ± 1.4	0.002
MCV, fL	88.3 ± 4.5	87.5 ± 4.6	0.001
Vitamin D, ng/mL	36.7 ± 13.5	34.1 ± 13.2	0.008
TG, mg/dL	70.8 ± 32.5	73.8 ± 35.2	0.009

Abbreviations: ALT, alanine aminotransferase; AST, aspartate aminotransferase; CPK, creatine phosphokinase; HDL, high-density lipoprotein cholesterol; MCV, mean corpuscular volume; TG, triglycerides; TSH, thyroid-stimulating hormone.

**Table 4:** Univariate logistic regression (EH as outcome).

Variable	OR	95% CI	*p*-Value	R^2^ (Nagelkerke)
Eosinophils	1.15	1.10–1.21	<0.0001	0.012
Creatinine	0.30	0.24–0.38	<0.0001	0.085
AST	1.04	1.03–1.05	<0.0001	0.048
ALT	1.03	1.02–1.04	<0.0001	0.031
CPK	1.001	1.000–1.002	<0.0001	0.018
HDL	1.02	1.01–1.03	<0.0001	0.022
TSH	0.90	0.84–0.96	0.002	0.009
MCV	1.02	1.01–1.03	0.001	0.010
Vitamin D	1.01	1.00–1.02	0.008	0.006
TG	0.99	0.98–1.00	0.009	0.007

Abbreviations: ALT, alanine aminotransferase; AST, aspartate aminotransferase; CI: confidence interval; CPK, creatine phosphokinase; EH: eccentric hypertrophy; HDL, high-density lipoprotein cholesterol; MCV, mean corpuscular volume; OR: odds ratio; TG, triglycerides; TSH, thyroid-stimulating hormone.

**Table 5:** Multivariate logistic regression in the overall population and stratified by sex (EH vs. NG).

	Overall		Men		Women	
Variable	OR (95% CI)	*p*-Value	OR (95% CI)	*p*-Value	OR (95% CI)	*p*-Value
**Age**	**1.04 (1.02–1.06)**	**<0.0001**	**1.04 (1.01–1.06)**	**0.011**	**1.05 (1.02–1.08)**	**0.002**
**Smoking**	**0.27 (0.16–0.47)**	**<0.0001**	**0.11 (0.04–0.30)**	**<0.0001**	**0.61 (0.31–0.88)**	**0.** **023**
**Creatinine**	0.28 (0.14–0.52)	<0.0001	**0.26 (0.20–0.36)**	**<0.0001**	**0.38 (0.28–0.54)**	**<0.0001**
**AST**	1.02 (1.01–1.03)	<0.0001	**1.06 (1.04–1.09)**	**<0.0001**	1.01 (0.99–1.02)	0.101
**Eosinophils**	1.12 (1.06–1.20)	<0.0001	1.07 (0.97–1.20)	0.067	**1.23 (1.10–1.39)**	**0.001**
HDL	1.02 (1.01–1.03)	<0.0001	1.01 (0.99–1.02)	0.210	1.03 (1.01–1.03)	0.015
TSH	0.94 (0.88–1.00)	0.052	0.99 (0.95–1.04)	0.367	0.92 (0.82–1.01)	0.074

Abbreviations: AST, aspartate aminotransferase; CI: confidence interval; EH: eccentric hypertrophy; HDL, high-density lipoprotein cholesterol; NG: normal geometry; OR: odds ratio; TSH, thyroid-stimulating hormone. Blue: men data; Pink: women data; Bold: statistically significant data.

## Data Availability

The data are not publicly available due to privacy or ethical restrictions. The data presented in this study are available on request from the corresponding author.

## Supplementary Materials

The following supporting information can be downloaded at https://www.mdpi.com/article/10.3390/medicina62071341/s1: Table S1. Distribution of sport disciplines according to sex within the eccentric hypertrophy (EH) and normal geometry (NG) groups. Table S2. Interaction analysis between sex and circulating biomarkers.
