# Supplementary material for: Biomarkers Identify Distinct Biological Signatures of Eccentric Hypertrophy in Elite Athletes: A Sex-Specific Analysis
Source: Medicina (Kaunas). 2026 Jul 11;62(7):1341. doi: 10.3390/medicina62071341 (PMC13413887; doi:10.3390/medicina62071341)
Supplement: Supplementary file 1 [file medicina-62-01341-s001.zip › medicina-4372317-supplementary.pdf]

Supplementary Table S1. Distribution of sport disciplines according to sex within the eccentric hypertrophy (EH) and normal geometry (NG) groups

EH athletes	Men (n = 317)	Women (n = 284)	p-value
Skill	9 (2.8%)	12 (4.2%)	
Power	61 (19.2%)	65 (22.9%)	
Mixed	77 (24.2%)	81 (28.5%)	
Endurance	170 (53.6%)	126 (44.3%)	**0.143**
**NG athletes**	**Men (n = 1070)**	**Women (n = 854)**	**p-value**
Skill	185 (17.3%)	127 (14.9%)	
Power	358 (33.5%)	301 (35.2%)	
Mixed	408 (38.1%)	339 (39.7%)	
Endurance	119 (11.1%)	87 (10.2%)	**0.416**

P-values were calculated using the χ^2 test.

Supplementary Table S2. Interaction analysis between sex and circulating biomarkers

Variable	Interaction p-value
Creatinine	0.643
AST	<0.001
Eosinophils	0.042
HDL	0.037
TSH	0.014

Interaction terms were evaluated by adding a sex × biomarker interaction term to the multivariable logistic regression model adjusted for age and smoking status.
